# Barium manganese(II) selenostannate(IV), BaMnSnSe_4_

**DOI:** 10.1107/S1600536811047283

**Published:** 2011-11-16

**Authors:** Abdeljalil Assoud, Bryan A. Kuropatwa, Holger Kleinke

**Affiliations:** a Department of Chemistry, Waterloo Institute for Nanotechnology, University of Waterloo, Waterloo, Ontario, Canada N2L 3G1

## Abstract

The title compound, BaMnSnSe_4_, was obtained by reaction of the elements at 1123 K in an evacuated silica tube. It adopts the BaCdSnS_4_ structure type, which is a variant of the SrIn_2_Se_4_ structure type. Its structure consists of distorted edge-sharing tetra­hedra, alternating with Mn and Sn atoms as central atom. These [MnSnSe_6_] units display corner sharing, forming stacked infinite layers in the *ac* plane. The three different Ba^2+^ atoms are located between the [MnSnSe_6_] layers, two on twofold rotation axes, and exhibit distorted square-antiprismatic coordinations.

## Related literature

For the synthesis and structures of quaternary sulfides *A* ^II^ *M* ^II^ *B* ^IV^S_4_ (*A* ^II^ = Ba, *M* ^II^ = Zn, Cd, Hg, Mn, *B* ^IV^ = Ge, Sn), see: Teske (1980*a* ▶,*b* ▶,*c* ▶,*d* ▶). For the SrIn_2_Se_4_ structure type, see: Eisenmann & Hofmann (1991 ▶) and for the BaCdSnS_4_ structure type, see: Assoud *et al.* (2004 ▶).

## Experimental

### 

#### Crystal data

BaMnSnSe_4_
*M* *_r_* = 626.81
Orthorhombic, 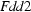

*a* = 22.3143 (10) Å
*b* = 22.7057 (11) Å
*c* = 13.4523 (6) Å
*V* = 6815.8 (5) Å^3^
*Z* = 32
Mo *K*α radiation
μ = 25.93 mm^−1^
*T* = 298 K
0.17 × 0.13 × 0.07 mm

#### Data collection

Bruker SMART APEX CCD diffractometer
Absorption correction: multi-scan (*SADABS*; Sheldrick, 1996 ▶) *T*_min_ = 0.67, *T*_max_ = 0.97
18387 measured reflections
6703 independent reflections
4800 reflections with *I* > 2σ(*I*)
*R*_int_ = 0.031

#### Refinement

*R*[*F* ^2^ > 2σ(*F* ^2^)] = 0.028
*wR*(*F* ^2^) = 0.072
*S* = 1.13
6703 reflections
129 parameters
1 restraint
Δρ_max_ = 1.95 e Å^−3^
Δρ_min_ = −1.64 e Å^−3^
Absolute structure: Flack (1983 ▶), 2823 Friedel pairs
Flack parameter: 0.044 (12)

### 

Data collection: *SMART* (Bruker, 2000 ▶); cell refinement: *SAINT* (Bruker, 1999 ▶); data reduction: *SAINT*; program(s) used to solve structure: *SHELXS97* (Sheldrick, 2008 ▶); program(s) used to refine structure: *SHELXL97* (Sheldrick, 2008 ▶); molecular graphics: *DIAMOND* (Brandenburg, 1999 ▶); software used to prepare material for publication: *SHELXL97*.

## Supplementary Material

Crystal structure: contains datablock(s) I, global. DOI: 10.1107/S1600536811047283/ru2017sup1.cif

Structure factors: contains datablock(s) I. DOI: 10.1107/S1600536811047283/ru2017Isup2.hkl

Additional supplementary materials:  crystallographic information; 3D view; checkCIF report

## Figures and Tables

**Fig. 1.**
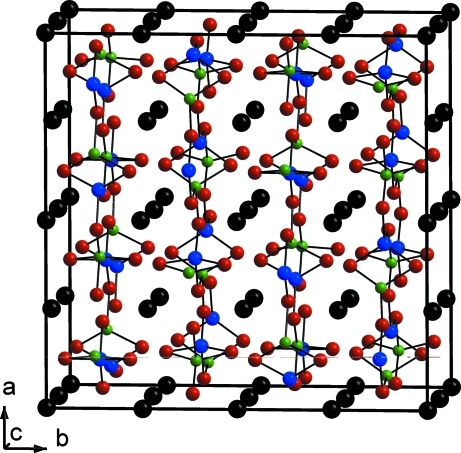
Crystal structure of BaMnSnSe4 in a projection along the c axis. Black: Ba; green: Mn; blue: Sn; red: Se.

**Fig. 2.**
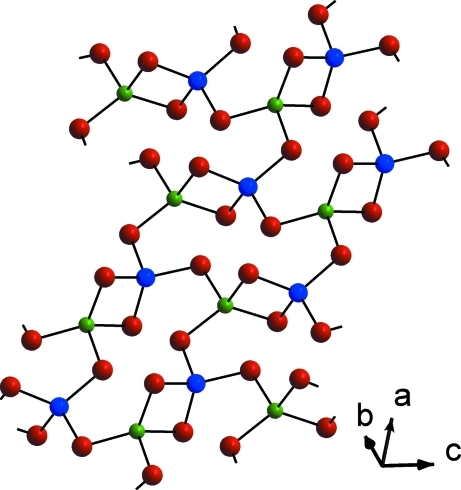
A single MnSnSe42- layer of the structure of BaMnSnSe4. Green: Mn; blue: Sn; red: Se.

## supplementary crystallographic information

### Comment

A number of quaternary sulfides *A*^II^*M*^II^*B*^IV^S_4_ (*A*^II^ = Ba, *M*^II^ = Zn, Cd, Hg, Mn, *B*^IV^ = Ge, Sn) were synthesized and reported in 1980 (Teske, 1980*a*,*b*,*c*,*d*). They adopt the BaCdSnS_4_ structure type and crystallize in the space group *Fdd*2, with the exception of BaHgSnS_4_, which adopts the *Pnn*2 group with the same structural motifs. These structures are variants of the SrIn_2_Se_4_ type (*Fddd*) (Eisenmann *et al.*, 1991) through the loss of the inversion centre due to the presence of two different *M*^II^ and *B*^IV^ centering cations. Here we report the synthesis and the crystal structure of the first selenide-based compound of this family, denoted BaMnSnSe_4_.

One dark red block-like single-crystal of BaMnSnSe_4_ was chosen under an optical microscope for study *via* single-crystal X-ray diffraction. The diffraction data were measured with the use of graphite-monochromated Mo—Kα radiation on a BRUKER Smart *APEX* CCD diffractometer. Data were collected by scans of 0.3° in two groups of 606 frames at φ = 0° and 90°. The exposure times per frame were 40 s. The data were corrected for Lorentz and polarization effects. Absorption corrections were based on fitting a function to the empirical transmission surface as sampled by multiple equivalent measurements using *SADABS*.

The unit cell refinement gave an orthorhombic *F*-centered cell with *a* = 22.3143 (10) Å, *b* = 22.7057 (11) Å, *c* = 13.4523 (6) Å, *V* = 6815.8 (5) Å^3^. The structure refinement was performed using the SrSn_2_Se_4_ model, producing satisfying residual factors. The Ba atoms replaced Sr atoms, the tetravalent Sn3 and Sn4 atoms remained in the same Wyckoff positions and the divalent Sn1 and Sn2 of SrSn_2_Se_4_ were reassigned as Mn1 and Mn2, while the Se positions were retained.

The new selenide, BaMnSnSe_4_ crystallizes in the BaCdSnS_4_ structure type, which - as previously mentioned - is a variant of the SrIn_2_Se_4_ structure type (Eisenmann *et al.*, 1991). We present just a short summary of the principal features of the structure since it has since been described elsewhere (Assoud *et al.*, 2004; Eisenmann *et al.*, 1991; Teske, 1980*a*,*b*,*c*,*d*). Fig. 1 shows a unit cell projection along the *c*-axis.

In this structure, there are four crystallographically independent metal atom sites, namely Mn1, Mn2, Sn3 and Sn4, which are each tetrahedrally coordinated by Se atoms. The tetrahedra are severely distorted, with Se—Mn—Se and Se—Sn—Se angles between 96° and 126°. The Mn—Se and Sn—Se bonds vary from 2.50 Å to 2.61 Å and 2.49 Å to 2.54 Å, respectively. Similar distortions were observed in the case of the mixed valent selenostannate SrSn_2_Se_4_, in SrMgSnSe_4_, (Assoud *et al.*, 2004) and in Ba*M*SnS_4_ (*M* = Mn, Zn, Cd, Hg) (Teske, 1980*a*,*b*,*c*,*d*), with Se—Sn—Se bond angles ranging from 94° to 129°. The tetrahedra in BaMnSnSe_4_ are interconnected by sharing edges between the MnSe_4_ and SnSe_4_ units. These [MnSnSe_6_] units then share corners with four identical units (two per tetrahedron) forming an infinite two-dimensional layer in the *ac* plane. The Ba atoms are in a square antiprismatic coordination, with Ba—Se bonds between 3.33 Å and 3.39 Å, occupying the space between the [MnSnSe_6_] layers. A single MnSnSe_4_^2-^ layer is displayed in Fig. 2.

### Experimental

The elements were acquired either in large blocks (Ba, 99.95% nominal purity, ALFA AESAR) or in powder form (Sn, 99.8%, -325 mesh, ALFA AESAR; Se, 99.5%, -100 mesh, Aldrich; Mn, 99.9%, -100 mesh, Aldrich). Because of the air sensitive nature of barium, all elements were handled in an argon-filled glove box. The elements were loaded in a silica tube (in 1:1:1:4 ratio), which was evacuated and sealed under dynamic vacuum, then placed into a temperature controlled resistance furnace. The silica tube was heated to 850°C within 24 h, kept at 850°C for a period of 4 days, and then cooled to 200°C within 8 days. Thereafter, the furnace was turned off. The samples looked homogeneous, comprising mostly microcrystalline red powder.

### Refinement

(type here to add refinement details)

### Crystal data

**Table:**

BaMnSnSe_4_	*D*_x_ = 4.887 Mg m^−^^3^
*M**_r_* = 626.81	Mo *K*α radiation, λ = 0.71073 Å
Orthorhombic, *F**d**d*2	Cell parameters from 1000 reflections
*a* = 22.3143 (10) Å	θ = 2.0–35°
*b* = 22.7057 (11) Å	µ = 25.93 mm^−^^1^
*c* = 13.4523 (6) Å	*T* = 298 K
*V* = 6815.8 (5) Å^3^	Block, red
*Z* = 32	0.17 × 0.13 × 0.07 mm
*F*(000) = 8544	

### Data collection

**Table:**

Bruker SMART APEX CCD diffractometer	6703 independent reflections
Radiation source: fine-focus sealed tube	4800 reflections with *I* > 2σ(*I*)
graphite	*R*_int_ = 0.031
ω scans	θ_max_ = 35.0°, θ_min_ = 2.0°
Absorption correction: multi-scan (*SADABS*; Sheldrick, 1996)	*h* = −35→35
*T*_min_ = 0.67, *T*_max_ = 0.97	*k* = −32→36
18387 measured reflections	*l* = −21→16

### Refinement

**Table:**

Refinement on *F*^2^	Secondary atom site location: difference Fourier map
Least-squares matrix: full	*w* = 1/[σ^2^(*F*_o_^2^) + (0.0177*P*)^2^ + 37.4914*P*] where *P* = (*F*_o_^2^ + 2*F*_c_^2^)/3
*R*[*F*^2^ > 2σ(*F*^2^)] = 0.028	(Δ/σ)_max_ = 0.001
*wR*(*F*^2^) = 0.072	Δρ_max_ = 1.95 e Å^−^^3^
*S* = 1.13	Δρ_min_ = −1.64 e Å^−^^3^
6703 reflections	Extinction correction: *SHELXL97* (Sheldrick, 2008), Fc^*^=kFc[1+0.001xFc^2^λ^3^/sin(2θ)]^-1/4^
129 parameters	Extinction coefficient: 0.0000363 (16)
1 restraint	Absolute structure: Flack (1983), 2823 Friedel pairs
Primary atom site location: structure-invariant direct methods	Flack parameter: 0.044 (12)

### Special details

**Table:**

Geometry. All e.s.d.'s (except the e.s.d. in the dihedral angle between two l.s. planes) are estimated using the full covariance matrix. The cell e.s.d.'s are taken into account individually in the estimation of e.s.d.'s in distances, angles and torsion angles; correlations between e.s.d.'s in cell parameters are only used when they are defined by crystal symmetry. An approximate (isotropic) treatment of cell e.s.d.'s is used for estimating e.s.d.'s involving l.s. planes.
Refinement. Refinement of *F*^2^ against ALL reflections. The weighted *R*-factor *wR* and goodness of fit *S* are based on *F*^2^, conventional *R*-factors *R* are based on *F*, with *F* set to zero for negative *F*^2^. The threshold expression of *F*^2^ > 2σ(*F*^2^) is used only for calculating *R*-factors(gt) *etc*. and is not relevant to the choice of reflections for refinement. *R*-factors based on *F*^2^ are statistically about twice as large as those based on *F*, and *R*- factors based on ALL data will be even larger.

### Fractional atomic coordinates and isotropic or equivalent isotropic displacement parameters (Å^2^)

**Table:**

	*x*	*y*	*z*	*U*_iso_*/*U*_eq_	
Ba1	0.5000	0.0000	0.51434 (7)	0.01461 (15)	
Ba2	0.2500	0.2500	0.76741 (7)	0.01464 (14)	
Ba3	0.498642 (19)	0.252102 (14)	0.51495 (6)	0.01478 (13)	
Mn1	0.42578 (5)	0.12560 (6)	0.80278 (9)	0.0199 (2)	
Mn2	0.38807 (5)	0.12366 (6)	0.26964 (9)	0.0198 (2)	
Sn3	0.36418 (2)	0.12530 (3)	0.51561 (4)	0.01241 (9)	
Sn4	0.56889 (2)	0.12516 (3)	0.22465 (4)	0.01295 (9)	
Se1	0.45804 (3)	0.12557 (5)	0.62286 (5)	0.01312 (14)	
Se2	0.37358 (3)	0.03939 (4)	0.89544 (8)	0.01486 (19)	
Se3	0.37316 (3)	0.21127 (4)	0.89774 (8)	0.0156 (2)	
Se4	0.28052 (3)	0.12657 (5)	0.63900 (6)	0.01672 (14)	
Se5	0.46781 (3)	0.12444 (5)	0.13819 (6)	0.01467 (15)	
Se6	0.37314 (4)	0.20988 (4)	0.39409 (7)	0.0160 (2)	
Se7	0.37225 (4)	0.03902 (4)	0.39730 (7)	0.01519 (19)	
Se8	0.54326 (3)	0.12649 (5)	0.40527 (6)	0.01468 (13)	

### Atomic displacement parameters (Å^2^)

**Table:**

	*U*^11^	*U*^22^	*U*^33^	*U*^12^	*U*^13^	*U*^23^
Ba1	0.0128 (3)	0.0114 (3)	0.0196 (4)	0.0003 (3)	0.000	0.000
Ba2	0.0142 (3)	0.0128 (3)	0.0169 (4)	0.0001 (3)	0.000	0.000
Ba3	0.0134 (2)	0.0122 (2)	0.0188 (4)	−0.0004 (2)	0.00082 (10)	−0.00034 (18)
Mn1	0.0209 (5)	0.0239 (6)	0.0148 (6)	−0.0002 (5)	0.0049 (4)	0.0000 (5)
Mn2	0.0183 (4)	0.0271 (6)	0.0141 (6)	−0.0004 (5)	0.0029 (4)	0.0001 (6)
Sn3	0.01249 (14)	0.0135 (2)	0.0113 (2)	0.00023 (18)	0.00052 (16)	0.0000 (2)
Sn4	0.01337 (17)	0.0137 (2)	0.0118 (2)	0.00020 (16)	0.00104 (15)	0.00006 (19)
Se1	0.0133 (2)	0.0150 (4)	0.0111 (4)	0.0002 (3)	0.0004 (2)	0.0001 (4)
Se2	0.0131 (4)	0.0128 (5)	0.0187 (5)	0.0012 (2)	−0.0017 (4)	−0.0018 (3)
Se3	0.0142 (4)	0.0134 (5)	0.0192 (5)	−0.0018 (2)	−0.0021 (4)	0.0019 (3)
Se4	0.0161 (3)	0.0176 (3)	0.0164 (4)	−0.0001 (3)	0.0056 (3)	0.0003 (4)
Se5	0.0129 (3)	0.0175 (4)	0.0136 (4)	−0.0002 (3)	0.0017 (2)	0.0000 (4)
Se6	0.0183 (5)	0.0133 (5)	0.0165 (5)	−0.0007 (3)	−0.0050 (4)	0.0024 (3)
Se7	0.0166 (4)	0.0129 (5)	0.0160 (5)	0.0007 (3)	−0.0039 (4)	−0.0021 (3)
Se8	0.0172 (2)	0.0150 (3)	0.0119 (3)	0.0007 (3)	0.0025 (2)	−0.0003 (4)

### Geometric parameters (Å, °)

**Table:**

Ba1—Se1^i^	3.3373 (11)	Mn1—Se3	2.6067 (17)
Ba1—Se1	3.3373 (11)	Mn1—Se4^ii^	2.6097 (13)
Ba1—Se8^i^	3.3664 (11)	Mn2—Se5	2.5086 (14)
Ba1—Se8	3.3664 (11)	Mn2—Se6	2.5974 (18)
Ba1—Se7	3.3748 (9)	Mn2—Se7	2.6014 (17)
Ba1—Se7^i^	3.3748 (9)	Mn2—Se8^vii^	2.6160 (14)
Ba1—Se6^ii^	3.3804 (10)	Sn3—Se4	2.4982 (8)
Ba1—Se6^iii^	3.3804 (10)	Sn3—Se6	2.5299 (12)
Ba2—Se5^iv^	3.3375 (12)	Sn3—Se7	2.5304 (11)
Ba2—Se5^v^	3.3375 (12)	Sn3—Se1	2.5431 (8)
Ba2—Se4	3.3619 (12)	Sn4—Se8	2.4963 (10)
Ba2—Se4^vi^	3.3620 (12)	Sn4—Se3^xi^	2.5276 (11)
Ba2—Se3^vi^	3.3764 (10)	Sn4—Se2^xi^	2.5277 (11)
Ba2—Se3	3.3765 (10)	Sn4—Se5	2.5377 (7)
Ba2—Se2^vii^	3.3838 (9)	Se2—Sn4^v^	2.5276 (11)
Ba2—Se2^viii^	3.3838 (9)	Se2—Ba3^iii^	3.3520 (10)
Ba3—Se5^ix^	3.3417 (12)	Se2—Ba2^ii^	3.3838 (9)
Ba3—Se1	3.3440 (11)	Se3—Sn4^v^	2.5275 (11)
Ba3—Se2^viii^	3.3521 (10)	Se3—Ba3^ix^	3.3705 (10)
Ba3—Se4^viii^	3.3587 (12)	Se4—Mn1^vii^	2.6097 (13)
Ba3—Se8	3.3619 (11)	Se4—Ba3^iii^	3.3586 (12)
Ba3—Se3^x^	3.3706 (10)	Se5—Ba2^xi^	3.3375 (12)
Ba3—Se6	3.3772 (10)	Se5—Ba3^x^	3.3416 (12)
Ba3—Se7^ii^	3.4132 (10)	Se6—Ba1^vii^	3.3804 (10)
Mn1—Se1	2.5251 (15)	Se7—Ba3^vii^	3.4133 (10)
Mn1—Se2	2.5965 (17)	Se8—Mn2^ii^	2.6160 (14)
			
Se1^i^—Ba1—Se1	128.12 (4)	Se1—Ba3—Se7^ii^	75.97 (2)
Se1^i^—Ba1—Se8^i^	62.758 (16)	Se2^viii^—Ba3—Se7^ii^	116.97 (3)
Se1—Ba1—Se8^i^	147.042 (13)	Se4^viii^—Ba3—Se7^ii^	130.54 (2)
Se1^i^—Ba1—Se8	147.044 (13)	Se8—Ba3—Se7^ii^	75.97 (2)
Se1—Ba1—Se8	62.757 (16)	Se3^x^—Ba3—Se7^ii^	68.02 (3)
Se8^i^—Ba1—Se8	128.32 (4)	Se6—Ba3—Se7^ii^	147.523 (18)
Se1^i^—Ba1—Se7	131.71 (2)	Se1—Mn1—Se2	126.01 (6)
Se1—Ba1—Se7	75.09 (2)	Se1—Mn1—Se3	126.75 (7)
Se8^i^—Ba1—Se7	77.20 (2)	Se2—Mn1—Se3	97.19 (5)
Se8—Ba1—Se7	79.33 (2)	Se1—Mn1—Se4^ii^	99.83 (4)
Se1^i^—Ba1—Se7^i^	75.10 (2)	Se2—Mn1—Se4^ii^	100.04 (5)
Se1—Ba1—Se7^i^	131.71 (2)	Se3—Mn1—Se4^ii^	101.51 (5)
Se8^i^—Ba1—Se7^i^	79.33 (2)	Se5—Mn2—Se6	122.69 (6)
Se8—Ba1—Se7^i^	77.20 (2)	Se5—Mn2—Se7	124.52 (6)
Se7—Ba1—Se7^i^	124.38 (4)	Se6—Mn2—Se7	96.54 (5)
Se1^i^—Ba1—Se6^ii^	77.07 (2)	Se5—Mn2—Se8^vii^	99.15 (5)
Se1—Ba1—Se6^ii^	76.82 (2)	Se6—Mn2—Se8^vii^	106.04 (5)
Se8^i^—Ba1—Se6^ii^	133.47 (2)	Se7—Mn2—Se8^vii^	106.12 (5)
Se8—Ba1—Se6^ii^	76.24 (2)	Se4—Sn3—Se6	118.66 (4)
Se7—Ba1—Se6^ii^	148.952 (15)	Se4—Sn3—Se7	118.69 (4)
Se7^i^—Ba1—Se6^ii^	67.98 (2)	Se6—Sn3—Se7	100.12 (4)
Se1^i^—Ba1—Se6^iii^	76.82 (2)	Se4—Sn3—Se1	103.79 (3)
Se1—Ba1—Se6^iii^	77.07 (2)	Se6—Sn3—Se1	107.44 (4)
Se8^i^—Ba1—Se6^iii^	76.24 (2)	Se7—Sn3—Se1	107.46 (3)
Se8—Ba1—Se6^iii^	133.47 (2)	Se8—Sn4—Se3^xi^	121.17 (4)
Se7—Ba1—Se6^iii^	67.98 (2)	Se8—Sn4—Se2^xi^	120.77 (4)
Se7^i^—Ba1—Se6^iii^	148.952 (15)	Se3^xi^—Sn4—Se2^xi^	101.07 (3)
Se6^ii^—Ba1—Se6^iii^	117.82 (4)	Se8—Sn4—Se5	104.04 (3)
Se5^iv^—Ba2—Se5^v^	121.73 (4)	Se3^xi^—Sn4—Se5	103.49 (4)
Se5^iv^—Ba2—Se4	155.821 (13)	Se2^xi^—Sn4—Se5	103.94 (4)
Se5^v^—Ba2—Se4	65.669 (17)	Mn1—Se1—Sn3	108.00 (4)
Se5^iv^—Ba2—Se4^vi^	65.669 (17)	Mn1—Se1—Ba1	119.97 (5)
Se5^v^—Ba2—Se4^vi^	155.821 (13)	Sn3—Se1—Ba1	88.90 (3)
Se4—Ba2—Se4^vi^	118.17 (4)	Mn1—Se1—Ba3	119.55 (5)
Se5^iv^—Ba2—Se3^vi^	72.66 (2)	Sn3—Se1—Ba3	88.79 (3)
Se5^v^—Ba2—Se3^vi^	78.02 (3)	Ba1—Se1—Ba3	117.91 (3)
Se4—Ba2—Se3^vi^	130.45 (2)	Sn4^v^—Se2—Mn1	80.81 (4)
Se4^vi^—Ba2—Se3^vi^	83.38 (2)	Sn4^v^—Se2—Ba3^iii^	91.25 (3)
Se5^iv^—Ba2—Se3	78.03 (3)	Mn1—Se2—Ba3^iii^	107.70 (4)
Se5^v^—Ba2—Se3	72.66 (2)	Sn4^v^—Se2—Ba2^ii^	113.64 (4)
Se4—Ba2—Se3	83.38 (2)	Mn1—Se2—Ba2^ii^	93.33 (3)
Se4^vi^—Ba2—Se3	130.45 (2)	Ba3^iii^—Se2—Ba2^ii^	149.93 (3)
Se3^vi^—Ba2—Se3	117.43 (4)	Sn4^v^—Se3—Mn1	80.62 (4)
Se5^iv^—Ba2—Se2^vii^	129.74 (2)	Sn4^v^—Se3—Ba3^ix^	114.00 (4)
Se5^v^—Ba2—Se2^vii^	80.38 (2)	Mn1—Se3—Ba3^ix^	91.78 (3)
Se4—Ba2—Se2^vii^	72.51 (2)	Sn4^v^—Se3—Ba2	91.42 (3)
Se4^vi^—Ba2—Se2^vii^	78.60 (2)	Mn1—Se3—Ba2	107.85 (4)
Se3^vi^—Ba2—Se2^vii^	69.03 (2)	Ba3^ix^—Se3—Ba2	150.40 (3)
Se3—Ba2—Se2^vii^	149.488 (14)	Sn3—Se4—Mn1^vii^	111.94 (4)
Se5^iv^—Ba2—Se2^viii^	80.38 (2)	Sn3—Se4—Ba3^iii^	118.05 (4)
Se5^v^—Ba2—Se2^viii^	129.74 (2)	Mn1^vii^—Se4—Ba3^iii^	92.00 (4)
Se4—Ba2—Se2^viii^	78.60 (2)	Sn3—Se4—Ba2	120.16 (4)
Se4^vi^—Ba2—Se2^viii^	72.51 (2)	Mn1^vii^—Se4—Ba2	93.59 (4)
Se3^vi^—Ba2—Se2^viii^	149.489 (15)	Ba3^iii^—Se4—Ba2	113.76 (3)
Se3—Ba2—Se2^viii^	69.03 (2)	Mn2—Se5—Sn4	107.90 (4)
Se2^vii^—Ba2—Se2^viii^	121.99 (4)	Mn2—Se5—Ba2^xi^	119.33 (5)
Se5^ix^—Ba3—Se1	124.48 (3)	Sn4—Se5—Ba2^xi^	92.14 (3)
Se5^ix^—Ba3—Se2^viii^	73.18 (2)	Mn2—Se5—Ba3^x^	120.96 (5)
Se1—Ba3—Se2^viii^	76.66 (2)	Sn4—Se5—Ba3^x^	91.31 (3)
Se5^ix^—Ba3—Se4^viii^	65.659 (18)	Ba2^xi^—Se5—Ba3^x^	114.86 (3)
Se1—Ba3—Se4^viii^	152.79 (2)	Sn3—Se6—Mn2	81.63 (4)
Se2^viii^—Ba3—Se4^viii^	83.91 (2)	Sn3—Se6—Ba3	88.28 (3)
Se5^ix^—Ba3—Se8	149.66 (2)	Mn2—Se6—Ba3	114.70 (4)
Se1—Ba3—Se8	62.735 (17)	Sn3—Se6—Ba1^vii^	118.29 (3)
Se2^viii^—Ba3—Se8	133.24 (2)	Mn2—Se6—Ba1^vii^	88.54 (4)
Se4^viii^—Ba3—Se8	123.35 (3)	Ba3—Se6—Ba1^vii^	147.80 (3)
Se5^ix^—Ba3—Se3^x^	80.50 (2)	Sn3—Se7—Mn2	81.55 (4)
Se1—Ba3—Se3^x^	130.17 (2)	Sn3—Se7—Ba1	88.28 (3)
Se2^viii^—Ba3—Se3^x^	150.829 (18)	Mn2—Se7—Ba1	112.79 (4)
Se4^viii^—Ba3—Se3^x^	73.79 (2)	Sn3—Se7—Ba3^vii^	118.84 (3)
Se8—Ba3—Se3^x^	75.68 (2)	Mn2—Se7—Ba3^vii^	88.13 (4)
Se5^ix^—Ba3—Se6	131.51 (2)	Ba1—Se7—Ba3^vii^	148.66 (3)
Se1—Ba3—Se6	74.95 (2)	Sn4—Se8—Mn2^ii^	112.78 (4)
Se2^viii^—Ba3—Se6	69.46 (3)	Sn4—Se8—Ba3	120.35 (4)
Se4^viii^—Ba3—Se6	80.52 (2)	Mn2^ii^—Se8—Ba3	89.00 (4)
Se8—Ba3—Se6	78.08 (2)	Sn4—Se8—Ba1	118.66 (4)
Se3^x^—Ba3—Se6	123.27 (4)	Mn2^ii^—Se8—Ba1	88.53 (4)
Se5^ix^—Ba3—Se7^ii^	77.86 (2)	Ba3—Se8—Ba1	116.59 (3)

Symmetry codes: (i) −*x*+1, −*y*, *z*; (ii) *x*+1/4, −*y*+1/4, *z*+1/4; (iii) −*x*+3/4, *y*−1/4, *z*+1/4; (iv) −*x*+3/4, *y*+1/4, *z*+3/4; (v) *x*−1/4, −*y*+1/4, *z*+3/4; (vi) −*x*+1/2, −*y*+1/2, *z*; (vii) *x*−1/4, −*y*+1/4, *z*−1/4; (viii) −*x*+3/4, *y*+1/4, *z*−1/4; (ix) −*x*+1, −*y*+1/2, *z*+1/2; (x) −*x*+1, −*y*+1/2, *z*−1/2; (xi) *x*+1/4, −*y*+1/4, *z*−3/4.
